# Topical 0.05% clobetasol cream in the treatment of chronic hand eczema: A protocol for systematic review and meta-analysis

**DOI:** 10.1097/MD.0000000000024418

**Published:** 2021-03-12

**Authors:** Mingyi Jing, Qianying Yu, Baohua Zhu, Fan Yuan, Jie Zhang, Li Peng, Wenxia Lin, Mingling Chen

**Affiliations:** Hospital of Chengdu University of Traditional Chinese Medicine, Chengdu, Sichuan Province, P. R. China.

**Keywords:** chronic hand eczema, meta-analysis, protocol, systematic review, topical 0.05% clobetasol cream

## Abstract

**Background:**

Chronic hand eczema (CHE) is a recurrent, frequently disabling skin condition that requires daily skin care to prevent transepidermal water loss, posing a significant burden of society and economy. In recent years, topical 0.05% clobetasol cream is widely used for the treatment of CHE for its efficacy, tolerability and safety. Whereas, no systematic review and meta-analysis has been updated up to now. Therefore, this work aims to assess the effectiveness and safety of topical 0.05% clobetasol cream in patients with CHE.

**Methods:**

Study on topical 0.05% clobetasol cream for CHE will be searched from their inception to December, 2020 with the language restrictions of English and Chinese in 8 databases (PubMed, Cochrane Library, Embase, the web of science, VIP, CNKI, CBM, and WAN FANG). According to the heterogeneity test, a fixed or random-effect model will be used to synthesize data. The primary outcome is the proportion of patients achieving more than 75% reduction in signs and symptoms according to the Hand Eczema Severity Index (HECSI). The secondary outcomes include: scored for 4 different characteristics of the lesions (redness, scaling, lichenification, and pruritus), QoL questionnaire, adverse events, and recurrence events. STATA 13.0 and Review Manager software 5.3 will be used for analysis and synthesis. Two or more reviewers will independently conduct the selection of studies, data extraction, and data analysis.

**Results:**

The results of the study expect to provide a high-quality, evidence-based recommendation on topical 0.05% clobetasol cream in the treatment of CHE for clinicians.

**Conclusion:**

The study will provide scientific and useful evidence for better use of topical 0.05% clobetasol cream in treating CHE.

**Ethics and dissemination:**

This study is a protocol for an overview of SRs/MAs that did not involve individual data. Thus, ethical approval is not required.

**OSF Registration number:**

DOI 10.17605/OSF.IO/SPHVZ

## 1 Introduction

Chronic hand eczema (CHE) is a chronic inflammation of the skin limited to the hands, which persists for 3 or more months or recurs 2 or more times within a 12-month time frame.^[1]^ Although most CHE patients have a disease from mild to moderate,^[2]^ the clinical processes are usually characterized by recurrent or persistent episodes for many years.^[3]^ It can be seen that exposure to atopic substances and atopic constitution are the main causes of CHE. CHE can be caused or aggravated by a variety of occupational exposures, including industry and health care workers.^[4–6]^ Some studies have suggested that nickel sulfate is an important allergen for aggravating hand eczema.^[7–12]^ In addition, about 1/3 to 1/2 of patients with CHE have a history of atopic disease or a family history of atopic disease, such as atopic dermatitis, allergic rhinitis, asthma, etc.^[7,13,14]^ Prevalence of CHE is higher in women than in men,^[15,16]^ and these people have a history of atopic dermatitis (AD).^[17]^ Therefore, patch tests are usually recommended for patients with CHE to detect allergens that they may be exposed to. Evidence also reveals that the economic burden of CHE is substantial and significant.^[18,19]^

At present, CHE has been treated in a variety of therapeutic approaches, including topical or oral corticosteroids, calcineurin inhibitor (tacrolimus, pimecrolimus), oral immunosuppressants (cyclosporin, mycofenolate mofetil, methotrexate), radiotherapy, phototherapy, topical or oral retinoids, barrier cream and gloves, emollients and antibacterial agent.^[20,21]^ However, it is particularly important to avoid exposure to allergens as soon as possible. Topical glucocorticoid preparation is the main treatment for eczema.^[22]^ Calcineurin inhibitors, such as tacrolimus or pimemox, may be considered to treat CHE when patients refuse glucocorticoids or are resistant to treatment with glucocorticoids. Studies have shown that the degree of side effects of pimecrolimus is less than that of tacrolimus.^[23,24]^ When there is no therapeutic benefit of topical medication, phototherapy, and oral retinoids, patients in CHE can also be treated with oral immunosuppressants, such as cyclosporine, methotrexate and azathioprine. Its effectiveness is mainly due to the good efficacy achieved in the treatment of atopic dermatitis by immunosuppressants.^[25–27]^ Although these treatments have a good immediate effect, there is a high recurrence rate of dermatitis after discontinuation of the treatment. Topical corticosteroids have been widely applied in hospital. The topical 0.05% clobetasol cream is the legal concentration approved for marketing by the Pharmaceutical Administration of the Ministry of Health and Welfare of Japan and the Food and Drug Administration of the United States (FDA), and this topical concentration has strong anti-inflammatory and anti-proliferative effects. Therefore, this systematic review and meta-analysis hope to further analyze the efficacy and safety of topical 0.05% clobetasol cream in the treatment of CHE.

## 2 Methods

### 2.1 Study registration

The protocol will be based on the preferred reporting items for systematic reviews and meta-analyses protocols guidelines.^[28]^ The OSF registration number is DOI 10.17605/OSF.IO/SPHVZ.

### 2.2 Eligibility criteria

#### 2.2.1 Type of studies

Our research will include only randomized controlled trials (RCTs).

#### 2.2.2 Type of participants

Our objective population will be patients who have chronic eczema with approximately similar severity in a hand or both hands, and also have their diseases longer than 3 months or have their diseases recurred 2 or more times within a 12-month time frame. The exclusion criteria for this study will be persons under the age of 18 years or over 65 years of age, lactating women, pregnant women, any related systemic diseases such as diabetes, hypertension, thyroid disease, kidney or liver disease, malignant tumor etc, and hypersensitivity to azathioprine.

#### 2.2.3 Type of interventions

Topical 0.05% clobetasol cream will be considered in the treatment group. The control group can include different concentrations of clobetasol, combined intervention (clobetasol plus other medicine), placebo, and any other active treatment.

#### 2.2.4 Types of outcome measures

##### 2.2.4.1 Primary outcome

Based on the Hand Eczema Severity Index (HECSI), the proportion of patients achieving more than 75% reduction in signs and symptoms is the primary outcome, which is considered as complete remission.^[29]^

##### 2.2.4.2 Secondary outcomes

The secondary outcomes cover scored for 4 different characteristics of the lesions including redness, scaling, lichenification, and pruritus.^[30]^ QoL questionnaire, adverse events, recurrence events are also included in the secondary outcomes.

### 2.3 Search methods for the identification of studies

#### 2.3.1 Information sources

We will perform a systematic clinical trial research search in databases of PubMed, Cochrane Library, Embase, the web of science, VIP, CNKI, CBM, and WAN FANG from their inceptions to December 2020 with a language limitation of English and Chinese. The key search terms of the study include “eczema” “chronic” “hand” “clobetasol” and “RCT”. The specific search strategy for the Cochrane Library can be seen in Table 1.

**Table 1:** The search strategy used in Medline (via Ovid). Table 1 search strategy for the Cochrane Library.

#1 MeSH descriptor: [eczema] explode all trees
#2 (“Eczemas”):ti,ab,kw or (“dermatitides, eczematous”):ti,ab,kw or (“eczematous dermatitides”):ti,ab,kw
#3 (“Dermatitis, eczematous”):ti,ab,kw or (“eczematous dermatitis”):ti,ab,kw”
#4 #1 or #2 or #3
#5 (“Chronic”):ti,ab,kw
#6 MeSH descriptor: [hand] explode all trees
#7 (“Hands”):ti,ab,kw
#8 #6 or #7
#9 #4 and #5 and #8
#10 MeSH descriptor:[clobetasol] explode all trees
#11 (“Cormax”):ti,ab,kw or (“temovate”):ti,ab,kw or (“clofenazon”):ti,ab,kw or (“Embeline E”):ti,ab,kw
#12 (“Clobetasol 17-Propionate”):ti,ab,kw or (“clobetasol 17 propionate”):ti,ab,kw or (“clobetasol propionate”):ti,ab,kw or (“OLUX”):ti,ab,kw
#13 (“Embeline”):ti,ab,kw or (“ Clobex”):ti,ab,kw or (“Dermovate”):ti,ab,kw
#14 #10 or #11 or #12 #13
#15 Mesh descriptor: [randomized controlled trial] explode all trees
#16 (“Randomized controlled trial”):ti,ab,kw or (“randomized”):ti,ab,kw or (“controlled”):ti,ab,kw
#17 #15 or #16
#18 #9 and #14 and #17

#### 2.3.2 Other resources

The reference lists of relevant RCTs and review articles related to topical 0.05% clobetasol cream in the treatment of CHE will be examined to identify potentially eligible studies. We also search for published journals and conference papers related to the topic, etc.

### 2.4 Collection and analysis of data

#### 2.4.1 Study selection

**Figure 1:**
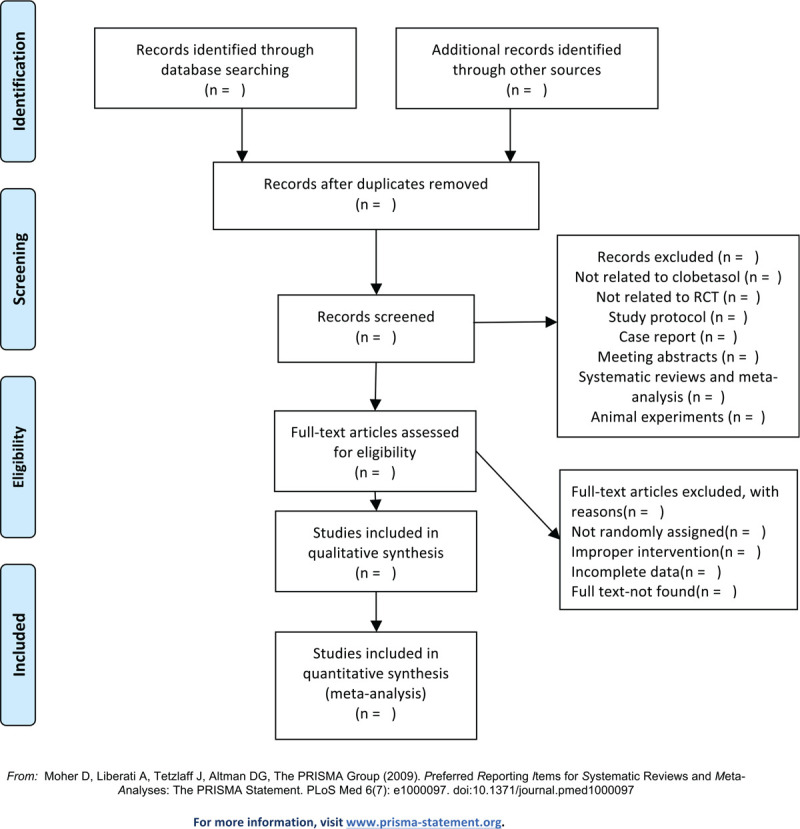
Flow diagram of the study selection process.

The selection of studies will be performed independently by 2 authors (FY and JZ). Then, EndNote software (X9) will be applied to manage the searched literature with the duplicate literatures excluded. Two authors will exclude irrelevant texts via reading the title and abstract, and the exclusion reasons will be recorded in the Excel table. The third investigators (QY and ML) will supervise the whole study selection process and resolve disagreements. The select study is shown in Figure 1.

#### 2.4.2 Data extraction

Two authors (MY and BH) will independently check the eligibility of the included studies and extract data. Extracted data will include general information (title, authors, country, publication year), methods (random method, blind method), participants (sex and age, number of participants), intervention group, control group, results (outcomes, adverse events), conclusions. During the data extraction process, any disagreement will be decided by the discussions between the third investigators.

#### 2.4.3 Risk assessment

The Risk of bias tool in the RevMan 5.3 software of the Cochrane Collaboration will be used independently by 2 authors (LP and WX) for quality assessment. Two authors will assess 7 aspects of each trial as follows: random sequence generation, allocation concealment, participant blinding, outcome evaluation blinding, incomplete outcome data, selective outcome reports, and other bias. The review results will be cross-checked and any disagreement will be arbitrated by the third investigators after consultation (QY and ML).

#### 2.4.4 Measures of therapeutic effect

Ninety-five percent confidence intervals will be used for continuous data to express the therapeutic effect. Risk ratio or odds ratio with 95% confidence intervals will be used to measure the therapeutic effect of dichotomous data.

#### 2.4.5 Dealing with insufficient data

We will contact the first author or corresponding author to get missing or insufficient data via telephone or email. If we fail to get insufficient data, we will analyze whether missing or insufficient data has an impact on the meta-analysis. The clinical trial study will be excluded with a large impact.

#### 2.4.6 Assessment of heterogeneity

Statistical heterogeneity will be assessed by the I^2^ statistic and Chi-squared test. It will show low or no heterogeneity with I^2^< 50%, and it will have significant heterogeneity among the trials with I^2^ ≥ 50%. If there is significant heterogeneity between the studies, we will explore the possible sources for heterogeneity with the sensitivity analysis or subgroup analysis.

#### 2.4.7 Data synthesis

RevMan V.5.3 software will be used for data synthesis. When there is no heterogeneity between studies (I^2^ < 50%), fixed effects model will be used for data synthesis. When the substantial heterogeneity is found (I^2^ ≥ 50%), random effects model will be used for data synthesis. If the data cannot be synthesized because of the substantial heterogeneity, descriptive analysis will be carried out.

#### 2.4.8 Subgroup analysis

If there are enough RCTs, we will perform subgroup analysis to find the source of heterogeneity by different study protocols, study quality, ethnicity, evaluation scale, etc.

#### 2.4.9 Sensitivity analysis

If there are insufficient samples, missing data, quality of analysis and research, methodological elements, sensitivity analysis will be performed. We will exclude the single study, and then analyze them again to evaluate the difference between the excluded results and the original combined results. However, the sensitivity analysis will not be performed if there is a high risk of bias in all included studies.^[31]^

#### 2.4.10 Evidence quality evaluation

The study will evaluate the quality of evidence of the merged results by the grading of recommendations assessment, development, and evaluation system. The quality of evidence will be divided into 4 levels: high, medium, low, and extremely low.^[32]^

## 3 Discussion

Given the huge financial burden and frequent recurrence of CHE treatment, which significantly affects patients’ life quality, it is necessary for both doctors and patients to have effective, safe, and economical treatment strategies. At present, topical 0.05% clobetasol cream has been widely used in the treatment of CHE due to its convenience and cheap. However, topical 0.05% clobetasol cream is not known to be effective and safe for CHE. Therefore, we aim at offering a relatively convincing conclusion on whether topical 0.05% clobetasol cream should be recommended as an adjunctive therapy for CHE via this systematic review. This study will be based on four parts: identification, research inclusion, data extraction, and data synthesis. If it is necessary to modify this protocol, modification's date, details and reasons will be provided. Besides, this review is limited to Chinese and English literatures due to language barriers.

## Author contributions

**Conceptualization:** Mingyi Jing.

**Data curation:** Mingyi Jing, Baohua Zhu, Fan Yuan, Jie Zhang, Li Peng.

**Formal analysis:** Baohua Zhu, Fan Yuan, Jie Zhang, Li Peng, Wenxia Lin.

**Methodology:** Mingyi Jing, Qianying Yu.

**Project administration:** Mingyi Jing, Mingling Chen, Wenxia Lin.

**Supervision:** Qianying Yu, Mingling Chen.

**Validation:** Mingyi Jing, Baohua Zhu, Fan Yuan.

**Writing – original draft:** Mingyi Jing.

**Writing – review & editing:** Mingyi Jing, Baohua Zhu, Qianying Yu.
